# Citrate anticoagulation versus systemic heparinisation in continuous venovenous hemofiltration in critically ill patients with acute kidney injury: a multi-center randomized clinical trial

**DOI:** 10.1186/s13054-014-0472-6

**Published:** 2014-08-16

**Authors:** Louise Schilder, S Azam Nurmohamed, Frank H Bosch, Ilse M Purmer, Sylvia S den Boer, Cynthia G Kleppe, Marc G Vervloet, Albertus Beishuizen, Armand RJ Girbes, Pieter M ter Wee, AB Johan Groeneveld, for the CASH study group

**Affiliations:** Department of Nephrology, VU University medical center, Amsterdam, De Boelelaan 1117, Amsterdam, 1081 HV The Netherlands; Departments of Intensive Care, Rijnstate hospital, Arnhem, The Netherlands; Haga hospital, Den Haag, The Netherlands; Spaarne hospital, Hoofddorp, The Netherlands; Medical center Alkmaar, Alkmaar, The Netherlands; VU University medical center, Amsterdam, The Netherlands; Erasmus medical center, Rotterdam, The Netherlands

## Abstract

**Introduction:**

Because of ongoing controversy, renal and vital outcomes are compared between systemically administered unfractionated heparin and regional anticoagulation with citrate-buffered replacement solution in predilution mode, during continuous venovenous hemofiltration (CVVH) in critically ill patients with acute kidney injury (AKI).

**Methods:**

In this multi-center randomized controlled trial, patients admitted to the intensive care unit requiring CVVH and meeting inclusion criteria, were randomly assigned to citrate or heparin. Primary endpoints were mortality and renal outcome in intention-to-treat analysis. Secondary endpoints were safety and efficacy. Safety was defined as absence of any adverse event necessitating discontinuation of the assigned anticoagulant. For efficacy, among other parameters, survival times of the first hemofilter were studied.

**Results:**

Of the 139 patients enrolled, 66 were randomized to citrate and 73 to heparin. Mortality rates at 28 and 90 days did not differ between groups: 22/66 (33%) of citrate-treated patients died versus 25/72 (35%) of heparin-treated patients at 28 days, and 27/65 (42%) of citrate-treated patients died versus 29/69 (42%) of heparin-treated patients at 90 days (P = 1.00 for both). Renal outcome, i.e. independency of renal replacement therapy 28 days after initiation of CVVH in surviving patients, did not differ between groups: 29/43 (67%) in the citrate-treated patients versus 33/47 (70%) in heparin-treated patients (P = 0.82). Heparin was discontinued in 24/73 (33%) of patients whereas citrate was discontinued in 5/66 (8%) of patients (P < 0.001). Filter survival times were superior for citrate (median 46 versus 32 hours, *P* = 0.02), as were the number of filters used (*P* = 0.002) and the off time within 72 hours (*P* = 0.002). The costs during the first 72 hours of prescribed CVVH were lower in citrate-based CVVH.

**Conclusions:**

Renal outcome and patient mortality were similar for citrate and heparin anticoagulation during CVVH in the critically ill patient with AKI. However, citrate was superior in terms of safety, efficacy and costs.

**Trial registration:**

Clinicaltrials.gov NCT00209378. Registered 13^th^ September 2005.

**Electronic supplementary material:**

The online version of this article (doi:10.1186/s13054-014-0472-6) contains supplementary material, which is available to authorized users.

## Introduction

Despite major improvements in therapy, the mortality rate for critically ill patients with acute kidney injury (AKI) remains 50% or higher [1]. Continuous venovenous haemofiltration (CVVH) is commonly used as renal replacement therapy in the ICU. The risk of clotting necessitates continuous anticoagulation for maintaining patency and function of the extracorporeal circuit and filter. Clotting and the resultant filter down-time adversely affect azotaemic control [2]. Excessive anticoagulation, however, may result in bleeding complications reported to occur in 10 to 50% of treatments [3]. Many anticoagulation methods have been pursued including low-dose heparin, low-molecular-weight heparin, prostanoids, mesilates and regional citrate anticoagulation. Heparin remains a commonly used anticoagulant for continuous renal replacement therapy. It is relatively easy to use and monitor and provides adequate extracorporeal anticoagulation. However, the risk of bleeding and development of heparin-induced thrombocytopenia (HIT) are important drawbacks.

By chelating calcium, citrate acts regionally as an anticoagulant when administered pre-filter and thereby reduces the risk of bleeding compared to systemic anticoagulation. Citrate is cleared by the tricarboxylic acid pathway in the liver, skeletal muscles and renal cortex producing bicarbonate. Citrate carries the risk of hypocalcaemia when it is insufficiently metabolized and thus accumulates. Also, metabolic acidosis may develop when citrate is insufficiently metabolized, while metabolic alkalosis develops when too much citrate enters the circulation and is subsequently metabolized. Moreover, when calcium-free replacement fluids are used, calcium supplementation is required. Citrate has been successfully adapted for use in continuous renal replacement therapies [4–9]. Several clinical trials comparing heparin to citrate for CVVH in critically ill patients have been published, yet most with small patient numbers and mainly focusing on filter survival times, with varying results [10–13]. In a recent study, low-molecular-weight heparin was compared to regionally administered citrate in post-dilutional CVVH. Interestingly, citrate-treatment reduced mortality which could be partly explained by less bleeding, suggesting improved biocompatibility for citrate as compared to heparin-based CVVH [14]. On the contrary, another recent trial comparing unfractionated heparin to citrate in predilutional CVVH did not show a survival benefit for citrate [15]. Two meta-analyses on this topic showed no difference in mortality, however, citrate reduced the risk of bleeding and was more efficacious in one of them [3,16].

The aim of this study was to compare regional citrate anticoagulation to systemic anticoagulation with unfractionated heparin during CVVH in a predilutional mode in terms of patient mortality, renal outcome, safety and efficacy. We hypothesized that regional citrate anticoagulation is a safe and efficient anticoagulant in critically ill patients with AKI requiring CVVH and favours renal and patient outcomes when compared to unfractionated heparin.

## Material and methods

### Study design

This was a multi-centre, randomized, non-blinded, prospective clinical trial with parallel group design performed in 10 ICUs in the Netherlands. The study was carried out in accordance with the Declaration of Helsinki, and was approved by the institutional review boards at each of the participating study centres (see Acknowledgements). The study was registered at clinicaltrials.gov number NCT00209378. Informed consent was obtained from all study participants or their next of kin. We use the acronym CASH for citrate anticoagulation versus systemic heparinisation.

### Patients

Adults admitted to the ICUs of the participating centres, and who required CVVH, were randomly assigned by sealed opaque envelopes with concealed treatment allocation inside, stratified by centre, and drawn by an independent individual not involved in the trial, to receive heparin or citrate for CVVH in predilution mode in a single-blinded fashion. All randomized patients were enroled. CVVH was started for AKI and uncontrolled uraemia, diuretic-resistant volume overload, respiratory distress, multiorgan failure, or any combination of these features, at the discretion of the treating physician and consulting nephrologist. Exclusion criteria were the presence of an increased bleeding risk (defined as a platelet count below 40 × 10^9^/L, an activated partial thromboplastin time (aPTT) longer than 60 seconds, a prothrombin time-international normalised ratio (PT-INR) greater than 2.0 or recent major bleeding), age below 18 or over 80 years, the need for therapeutic systemic anticoagulation (heparin or coumarins) or a known HIT. The administration of activated protein C or plasma exchange therapy were also considered exclusion criteria, as was chronic dependence on renal replacement therapy prior to admission to the ICU. The presence of liver disease of any kind was not an exclusion criterion.

### Study protocol

The participating centres used the locally available haemofiltration machine, venous catheter and haemofilter. In patients receiving heparin, commercially prepared bicarbonate-buffered haemofiltration replacement solution (HF32bic, Dirinco, Rosmalen, the Netherlands) was used in most patients; lactate-based (BH504, Dirinco) haemofiltration replacement solution was used in six patients. For citrate anticoagulation, we adapted the protocol conceived by Pallson and Niles [6], and used a custom-made calcium-free trisodium citrate replacement fluid (HFCitPre, Dirinco), acting as anticoagulation and buffer in predilution mode, as described before [17,18]. For composition of the replacement fluids used, see Additional file 1. Patients anticoagulated with heparin received a heparin bolus of 5,000 IU in a pre-filter fashion at the arterial pole prior to the start of CVVH and a separate heparin pump (20,000 IU/48 mL) was set at 2.0 mL/h and adjusted targeting a systemic aPTT of 50 seconds. Patients anticoagulated with regional citrate had a separate intravenous calcium drip, with calcium glubionate (Calcium Sandoz, containing 0.225 mmol/mL calcium, Novartis Consumer Health, Breda, The Netherlands). Calcium administration was adapted to concentrations of systemic ionised calcium by a specially designed algorithm, targeting systemic ionised calcium levels of 1.0 to 1.35 mmol/L [19]; for calcium pump settings see Additional file 1. Calcium levels in the extracorporeal circuit were not measured routinely, in order to keep the treatment protocol simple. Blood flow was initially set at 180 mL/minute in both groups. Predilution replacement flow rates varied between 2,000 and 4,000 mL/h according to local guidelines. The rate of infusion of replacement solution was coupled to the blood flow, aiming at stable citrate concentrations in the extracorporeal circuit. The replacement solution ran through a warming coil set at 39°C.

The pH, anion gap, ionised and total calcium and their ratio were monitored in patients on citrate, at least four times daily. The first measurement was done 1 h after initiation of CVVH. Routine daily laboratory measurements included acid-base balance, electrolytes, haemoglobin, and white blood cell and platelet counts. Also, levels of magnesium and sodium were monitored to detect citrate-related derangements. In patients on heparin the aPTT was determined every 6 to 8 h. An electrocardiogram was made if hypocalcaemia (ionised calcium below 0.9 mmol/L) occurred. Citrate accumulation was suspected if the patient fulfilled one or more of the following criteria: a ratio of total calcium to ionised calcium greater than 2.5, clinical signs of hypocalcaemia (tetanic symptoms or prolonged QT interval not due to medication), or progressive acidosis (pH <7.20) with an increased anion gap (>13 mmol/L) in the presumed absence of anions other than citrate. If there were signs of citrate accumulation, CVVH was continued with heparin. Patients treated with heparin, who were clinically suspected of having developed a HIT, continued CVVH with citrate. Also, in case of a clinically suspected bleeding episode, heparin was discontinued and patients continued CVVH with regional citrate anticoagulation. The crossover between study arms was documented. Filters were routinely replaced after 72 hours of use, apart from incidental cases when still functioning well (n = 2). CVVH was continued at the discretion of the treating physician. Patients were otherwise treated by board-certified intensivists, according to international and national guidelines.

### Study endpoints

Primary endpoints were patient mortality at 28 and 90 days after initiation of CVVH and renal outcome. For renal outcome, independence of renal replacement therapy 28 days after initiation of CVVH in surviving patients was studied. Secondary endpoints were safety and efficacy. Safety was defined as the absence of any adverse event necessitating discontinuation of study anticoagulant, such as bleeding or HIT in the heparin group and citrate accumulation in the citrate group. For efficacy, the survival time of the first filter, the number of filters used and off-time within the first 72 hours of therapy were studied. Additionally, a cost analysis for the first 72 hours of prescribed CVVH was performed. Follow up until day 90 was achieved in 134 of the 139 (96%) patients.

### Data collection

Severity of illness and organ failure were scored using the acute physiology and chronic health evaluation II (APACHE II) and the sequential organ failure assessment (SOFA) on days 0 and 3. All data were prospectively collected by the local researcher in the patient data management system of the participating centre. A research nurse, not involved in patient care, was responsible for the coordination of the collection of case record forms and accuracy of the database. The etiology of AKI, categorised as ischaemic, due to sepsis or other (including contrast nephropathy and rabdomyolysis), was assessed by the treating physician. The costs during the first 72 hours of prescribed CVVH were calculated using the following parameters: cost of replacement solution used (during first 72 hours of therapy minus off-time), cost of heparin administered in the heparin group, cost of calcium administered in the citrate group, costs of total numbers of filter sets used and the half-hourly wage of nursing staff per filter change.

### Statistical analysis

We hypothesized an absolute mortality reduction of 15% after 28 days for patients on citrate, as compared to that of patients on heparin, with an estimated mortality rate of 65%. The power of the study was set to be 90% at 5% significance, so that the sample size was targeted at 180 patients per group. Values are presented as the median and range, because most data were non-normally distributed (Kolmogorov-Smirnov test, *P* <0.05). We compared groups using the Mann-Whitney *U*-test, the chi square (X^2^) test, or the Fisher exact test, where appropriate. Patient and filter survival data were analysed using Kaplan-Meier plots and log rank testing. Data were analysed on an intention-to-treat basis. We also performed a per-protocol analysis, excluding patients who switched from the assigned anticoagulation. Backward stepwise multiple logistic regression was used to evaluate the contribution of age, disease severity, prescribed dose and anticoagulation regimen on mortality at 28 days. The Hosmer-Lemeshow test was used to verify adequate calibration, as indicated by a *P*-value >0.05. Additionally, univariate logistic regression was performed generating odds ratios for mortality at 28 days in the following subgroups: age (higher versus lower than the median), APACHE II scores (higher versus lower than the median), prescribed dose (higher or lower than the recommended 20 mL/kg/h), sepsis versus non-sepsis and circulatory or respiratory failure at ICU admission. A two-sided *P*-value <0.05 was considered statistically significant; exact *P*-values are given unless <0.001.

## Results

From April 2005 until March 2011, 139 patients were randomly assigned to citrate or heparin. The Additional files present number of patients included by the participating centres (see Additional file 2) and a consort diagram (see Additional file 3). Baseline and CVVH characteristics were similar between groups, apart from more frequent respiratory failure on admission in the heparin group (Table 1). In the citrate group, 26 of the 66 patients (39%) had sepsis versus 26 of the 73 patients (36%) in the heparin group (*P* = 0.73). An aPTT >50 sec was achieved in 136/247 measured time points (55%) for the first filter in the heparin group. The study was prematurely discontinued because of slow enrolment of patients.

**Table 1:** **Baseline characteristics**

	**Citrate (n = 66)**	**Heparin (n = 73)**	***P*** **-value**
Age, years	67 (36 to 87)	67 (23 to 85)	0.51
Male sex	44 (67)	49 (67)	1.00
Weight, kg	85 (50 to 180)	80 (40 to 220)	0.54
Reason for admission to ICU			0.02
Respiratory failure	23 (35)	45 (62)	
Circulatory failure	18 (27)	10 (14)	
Trauma	2 (3)	2 (3)	
Post-CPR	4 (6)	1 (1)	
Postoperative	19 (29)	15 (21)	
Cause of acute kidney injury			0.64
Sepsis	27 (41)	27 (37)	
Ischaemic	33 (50)	37 (51)	
Other	6 (10)	9 (13)	
**At start of CVVH:**			
APACHE II	23 (11 to 53)	25 (6 to 43)	0.10
SOFA	10 (2 to 19)	11 (3 to 18)	0.45
SOFA, day 3	9 (2 to 20)	9 (1 to 16)	0.82
Mechanical ventilation	45 (81)	55 (81)	1.00
Vasopressor dependency	41 (75)	54 (79)	0.53
Length of stay in ICU, days	2 (0 to 30)	2 (0 to 25)	0.37
Creatinine, μmol/L	326 (54 to 704)	325 (56 to 845)	0.66
Urea, mmol/L	20.1 (3.3 to 54)	23.6 (4 to 64)	0.38
Potassium, mmol/L	4.6 (2.4 to 8.8)	4.6 (2.9 to 6.5)	0.83
Diuresis in 24 h prior to CVVH, mL	360 (0 to 3,975)	421 (0 to 2745)	0.65
Prescribed dose, mL/kg/h	21 (8 to 32)	23 (7 to 35)	0.18

Median (range) or number (percentage) where appropriate. CVVH, continuous venovenous haemofiltration; CPR, cardiopulmonary resuscitation; APACHE-II, acute physiology and chronic health evaluation score; SOFA, sequential organ failure assessment.

### Primary outcome measures

**Figure 1:**
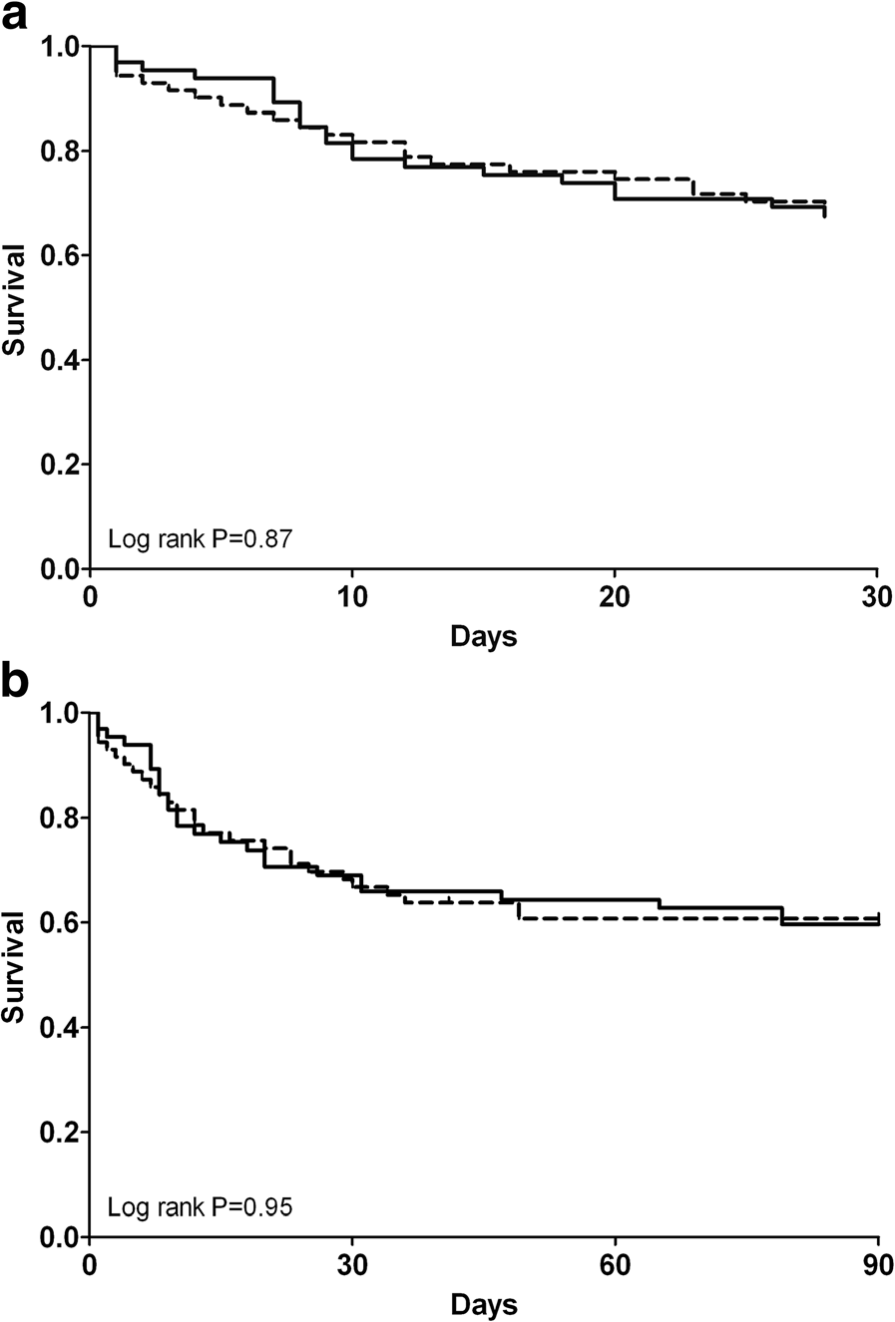
**Patient survival. (a)** Survival 28 days after initiation of continuous venovenous haemofiltration (CVVH). **(b)** Survival 90 days after initiation of CVVH. Continuous line represents citrate, dotted line represents heparin.

Mortality rates at 28 and 90 days did not differ between groups: 22/66 citrate patients (33%) died versus 25/72 patients (35%) in the heparin group at 28 days, and 27/65 citrate patients (42%) died versus 29/69 patients (42%) in the heparin group at 90 days (*P* = 1.00 for both). Indeed, survival curves for 28- and 90-day outcomes were similar among anticoagulation groups (Figure 1). Also, a per-protocol analysis did not show any difference in mortality at 28 and 90 days: 18/61 citrate patients (30%) died versus 15/48 patients (31%) in the heparin group at 28 days and 23/60 citrate patients (38%) died versus 18/47 patients (38%) in the heparin group at 90 days (*P* = 1.00 for both). Multiple logistic regression showed higher age and high APACHE II scores at admission to be independent predictors of mortality at 28 days (*P* = 0.05 for both), whereas prescribed dose and anticoagulation regimen were not. In univariate logistic regression, there was no benefit from citrate anticoagulation in terms of mortality reduction at 28 days in any of the defined subgroups (see Additional file 4). Concerning renal outcome, there was no difference between the groups in dialysis independence among surviving patients 28 days after start of CVVH, with 29/43 patients (67%) in the citrate group versus 33/47 patients (70%) in the heparin group (*P* = 0.82).

### Secondary outcome measures

Adverse events necessitating discontinuation of the primary assigned anticoagulant occurred with heparin more frequently than with citrate (Table 2 and Additional file 3). In one patient, citrate was discontinued and anticoagulation withheld. Citrate accumulation was suspected in 5 of the 66 patients (8%) randomized to citrate and proven in 4 patients (6%); in 2 patients there was a persistently elevated anion gap, attributed to citrate accumulation, and in 2 patients the calcium ratio exceeded 2.5 after 60 and 72 hours of therapy. Finally, one patient was erroneously ascribed to citrate accumulation. Of all measured ionised calcium levels within 72 hours in the citrate group, 61/508 (12%) were <0.9 mmol/L and none of the measurements exceeded 1.35 mmol/L. Potential treatment related derangements, such as metabolic alkalosis, hypernatremia or hypomagnesemia did not occur more often in the citrate group than in the heparin group (Table 2). Clinically suspected HIT was reported in 6 of 73 patients (8%) on heparin. There was a trend for fewer bleeding episodes in the citrate group (n = 5 versus n = 10 in the heparin group, *P* = 0.08), however, this did not result in a difference between groups in patients needing >2 erythrocyte concentrates (2 versus 4 patients in the heparin group, *P* = 0.68).

**Table 2:** **Secondary outcomes**

	**Citrate (n = 66)**	**Heparin (n = 73)**	***P*** **-value**
**Safety,** **discontinuation of study anticoagulant**			
Within 72 h	2 (3)	9 (12)	0.06
Bleeding episode	0	2 (22)	
HIT	0	2 (22)	
Frequent filter failure	0	3 (33)	
Citrate accumulation	2 (100)	0	
Miscellaneous	0	2 (22)	
Within 28 days	5 (8)	24 (33)	<0.001
Bleeding episode	0	8 (33)	
HIT	0	6 (25)	
Frequent filter failure	0	7 (29)	
Citrate accumulation	4 (80)	0	
Miscellaneous	1 (20)	3 (13)	
Bleeding episode within 28 days	3 (5)	10 (14)	0.08
Requirement of >2 packed cells	2 (3)	4 (6)	0.68
**Metabolic derangements**, **during first 72 hours of therapy**			
pH >7.50	1 (2)	0	1.00
Sodium >150 mmol/L	4 (7)	3 (5)	0.71
Magnesium <0.7 mmol/L	8 (15)	6 (9)	0.40
**Efficacy, intention to treat**			
Survival time first filter, h	46 (1 to 138)	32 (1 to 72)	0.02
Number of filters used within 72 h	1 (1 to 5)	2 (1 to 9)	0.002
Off-time within 72 h, h	1 (0 to 12)	3 (0 to 31)	0.002
Reason for circuit disconnection			0.01
Circuit clotting	16 (24)	35 (51)	
Elective filter change (72 h)	20 (30)	6 (9)	
Catheter dysfunction	4 (6)	8 (12)	
Termination of CVVH^1^	10 (15)	10 (12)	
Transport	4 (6)	1 (1)	
Technical problems	8 (12)	5 (7)	
Therapy change^2^	2 (3)	3 (4)	
Miscellaneous	2 (3)	1 (1)	
Total duration of CVVH, h	123 (4 to 999)	73 (5 to 672)	0.18
**Efficacy, per protocol**	n = 61	n = 49	
Number of filters used within 72 h	1 (1 to 5)	2 (1 to 9)	0.04
Off-time within 72 h, h	2 (0 to 12 )	0 (0 to 31)	0.01
Total duration of CVVH, h	117 (4 to 999)	70 (5 to 672)	0.04
**Costs**			
Total cost of first 72 h of CVVH, €	553 (436 to 872)	663 (320 to 1,319)	<0.001
Replacement fluid, €	316 (225 to 366)	429 (119 to 736)	<0.001
Wage nursing staff for filter change, €	19 (19 to 95)	38 (19 to 171)	0.02
Filter sets, €	85 (85 to 425)	170 (85 to 765)	0.02
Heparin, €	0	6.46 (3.84 to 6.74)	<0.001
Calcium glubionate, €	82 (70 to 84)	0	<0.001

Median (range) or number (percentage), where appropriate. HIT, heparin-induced thrombocytopenia; CVVH, continuous venovenous haemofiltration; € = euro. ^1^Treatment withdrawal or renal function recovery.

^2^Continuous renal replacement therapy to intermittent renal replacement therapy.

**Figure 2:**
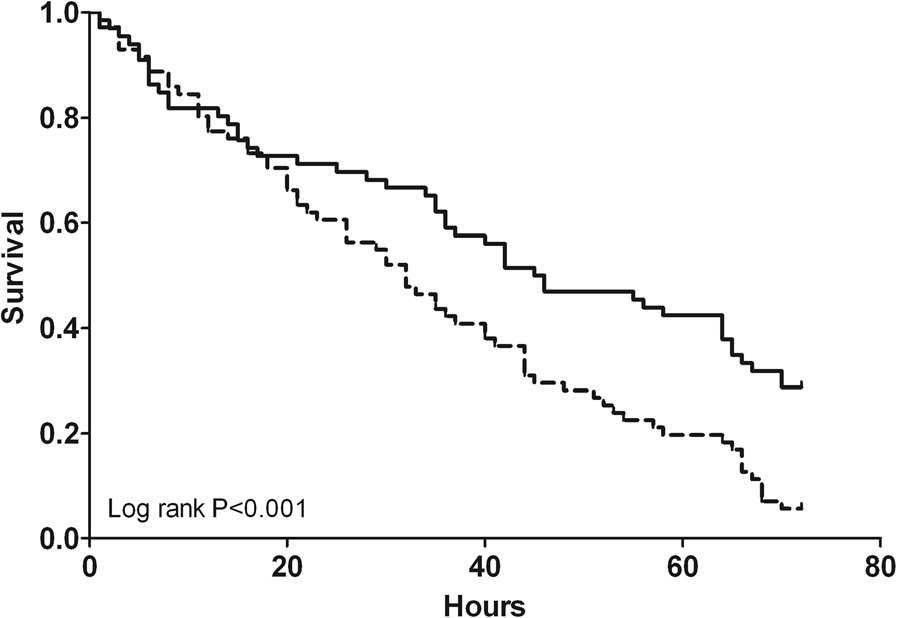
**Survival times for the first filter.** Continuous line represents citrate, dotted line represents heparin.

Efficacy parameters suggested superiority for citrate (Table 2). Survival times of the first filter were superior for citrate, with survival curves shown in Figure 2. An analysis excluding patients in whom the filter ran for more than 72 hours did not differ from the presented results. Furthermore, the off-time within 72 hours was less with citrate (1 (0 to 12) h versus 3 (0 to 31) h for heparin, *P* = 0.002), as were the number of filters used (1 (1 to 5) versus 2 (1 to 9) for heparin, *P* = 0.002). In per-protocol analysis the total duration of CVVH was longer in the citrate group, at 117 hours versus 70 hours for heparin (*P* = 0.04). There was a higher incidence of circuit disconnection due to clotting of the circuit in the heparin group (51% versus 24% in the citrate group) and more elective filter changes in the citrate group (30% versus 9% in the heparin group, *P* = 0.01). The total costs during the first 72 hours of prescribed CVVH were lower in citrate-based CVVH (*P* <0.001, Table 2.)

## Discussion

The present multi-centre randomized controlled trial in critically ill patients with AKI suggests that regional anticoagulation with citrate for CVVH is superior to heparin in terms of safety, efficacy, and costs, but not in terms of renal and patient outcomes. Mortality rates 28 and 90 days after initiation of CVVH did not differ between the two anticoagulation regimens.

Independency of dialysis at day 28 was achieved in about 70% of surviving patients either anticoagulated with citrate or with heparin. These numbers are low compared to those observed by some [14,20], yet similar to numbers described by others [21]. We evaluated renal function recovery at 28 days after start of CVVH, while later recovery is still possible. Also, in our study there was less AKI due to sepsis and more due to ischaemic events in comparison to the trial reported by Oudemans *et al*. [14], and an ischaemic aetiology may carry a worse renal prognosis.

We powered the study for approximately 360 patients on the basis of 28-day mortality; however enrolment was slower than anticipated, mostly due to the need for therapeutic systemic anticoagulation or the development of increased bleeding risk. This resulted in premature discontinuation of the study after 6 years of enrolment. The most substantial difference in mortality observed was 2% in favour of citrate at 28 days. In hindsight, this difference to become statistically significant at the 5% level would have necessitated 9,600 patients per group for 90% power. Also, in the initial power analysis we used an estimated mortality rate of 65% at 28 days in the heparin group, but the observed mortality rate was substantially lower. This tendency for improving AKI outcomes over the years has been noted by others [22].

To our knowledge, this is the second randomized clinical trial comparing vital outcome in citrate and heparin-based CVVH in predilutional mode, using citrate-containing replacement fluid [15]. Most trials comparing heparin to citrate in CVVH had small numbers of patients and mainly focused on filter survival [10–13], and only two had mortality as an outcome measure [14,15]. Our findings are in concordance with a recent trial comparing systemic heparin to regional citrate [15], where 28-day mortality was similar for both groups (47% mortality for citrate, 41% for heparin at 28 days). However, our results are in contrast with a trial comparing the low molecular weight heparin nadroparin to systemically administered citrate in a post-dilution fashion [14], where citrate reduced mortality (90-day mortality 45% for citrate, 62% for nadroparin). Mortality rates in the present study were somewhat lower than in previous studies and this could, at least in part, be attributed to a lower frequency of sepsis in our patient group. Also, APACHE-II scores at baseline, and age, were higher as reported by Oudemans *et al*. [14], yet similar to those reported by Hetzel *et al*. [15]. We could not confirm the beneficial effect on mortality rates in cases of sepsis that were suggested by Oudemans *et al*.; survival rates were similar for septic and non-septic patients and the anticoagulation used did not affect this number. Moreover, the lack of benefit for citrate regarding mortality persisted in subgroup analyses.

Citrate was safer than heparin in terms of less need for discontinuation of the anticoagulant. Citrate accumulation was present in 6% of patients. The frequency of accumulation in our study is similar to rates reported by others [17,18]. The incidence of clinically suspected HIT (6/73 patients (8%) in the heparin group) was similar to numbers reported by Hetzel *et al*., yet higher than numbers reported by others [23,24]. When HIT was suspected, the diagnosis was not routinely confirmed by antibody testing, so that the incidence may have been overestimated.

Citrate was superior to heparin in terms of efficacy. The filter survival time of the first filter used was superior for citrate compared to heparin, supporting the beneficial effect of citrate in this respect as described by others [15–17]. Improved filter survival implicates less off-time, which indeed was the case in our study for citrate. An aPTT >50 seconds, which we used as indication of adequate anticoagulation during heparin treatment, was achieved in 55% of the measurements of the first filter. This could partly explain the difference in filter survival, even though some have questioned whether aPTT is a determinant of filter survival [25,26]. Besides, heparin was administered in a pre-filter fashion, therefore systemically measured aPTTs could underestimate actual aPTT in the extracorporeal circuit [25]. Also, there seems to be a range of aPTT targets during CVVH in the literature, where some target a lower aPTT of 40 to 45 seconds [25]. This study demonstrates clinical practice, where anticoagulation using heparin is challenging due to problems such as heparin resistance and an increased bleeding risk when administering high doses of heparin, especially in critically ill patients [27–29]. Nevertheless, the survival times of the first filter in the heparin group were similar to those described by others [10–13,15]. Additionally, the costs of the first 72 hours of prescribed CVVH were lower in citrate-based CVVH, which can be attributed to lower costs of filter sets and less labour due to fewer filters used during treatment with citrate, adding another argument in favour of citrate as compared to heparin.

Limitations of the present study include the long inclusion period, because enrolment was slower than anticipated. Subsequently, the study was prematurely discontinued and therefore underpowered for mortality. We cannot exclude that the availability of citrate-based CVVH and positive experiences concerning filter survival by physicians and nursing staff raised the threshold for enrolling patients in the study and thus resulted in selection bias. An envelope-derived method for randomization may be inferior to a computer-based method. There was a substantial range in the prescribed dose and in some patients recommended doses exceeding 20 mL/kg/h were not achieved. However, there was no difference in the prescribed dose between groups nor did the prescribed dose contribute to mortality. There was a slight imbalance between groups in reasons for admission to the ICU, but there were no differences regarding anticoagulation and mortality between admission categories. Though a beneficial effect of one biocompatible filter over the other has not been demonstrated, we cannot rule out that some bias is introduced, as the centres utilized different filters.

The total duration of CVVH was longer in the citrate group in a per protocol analysis. We hypothesize that this is due to longer filter survival with citrate, since it is common practice to postpone termination of CVVH until circuit failure is impending. Although citrate and heparin treatment carried similar mortality rates during CVVH, the improved filter survival, less need for discontinuation of the anticoagulant due to adverse events and lower costs are substantial advantages of citrate and should be considered when deciding on which anticoagulation in CVVH is best for critically ill patients with AKI. Our results support current guidelines on anticoagulation in CVVH, suggesting citrate rather than heparin for anticoagulation in patients without contraindications for citrate [30].

## Conclusions

In conclusion, renal outcome and patient mortality were similar for citrate- and heparin-based anticoagulation during CVVH in the critically ill patient with AKI, in this randomized multi-centre clinical trial. However, citrate had advantages in terms of safety, efficacy and costs and should therefore be considered as first choice for anticoagulation in CVVH.

## Key messages

- Patient mortality and renal outcome were similar for citrate and heparin anticoagulation during CVVH in the critically ill patient with AKI
- Citrate was safer than heparin in terms of less need for discontinuation of the anticoagulant due to adverse events during CVVH
- Citrate was superior to heparin in terms of efficacy demonstrated by improved filter survival and less off-time during CVVH
- The costs of the first 72 hours of prescribed CVVH were lower in citrate-based CVVH as compared to heparin-based CVVH.

## Additional files

Additional file 1:
**Composition of replacement fluids and calcium pump settings.** Composition of the replacement fluids used, the rate of infusion of replacement solution coupled to the blood flow and the protocol for calcium pump adjustments during citrate-based continuous venovenous haemofiltration (CVVH). Additional file 2:
**List of participating centres and number of patients included.** The number of patients included by participating centres. Additional file 3:
**Consort diagram.** Consort diagram demonstrating eligible patients. Also, crossovers between anticoagulation groups are listed. Additional file 4:
**Mortality in subgroups at 28 days.** Mortality at 28 days for citrate and heparin in the following subgroups: age (higher versus lower than median), acute physiology and chronic health evaluation (APACHE) II scores (higher versus lower than the median), prescribed dose (higher or lower than the recommended 20 mL/kg/h), sepsis versus non-sepsis and circulatory or respiratory failure at ICU admission.

## Abbreviations

AKI: acute kidney injury
APACHE II: acute physiology and chronic health evaluation
aPTT: activated partial thromboplastin time
CVVH: continuous venovenous haemofiltration
HIT: heparin-induced thrombocytopenia
PT-INR: prothrombin time-international normalised ratio
SOFA: sequential organ failure assessment

## References

[1] Uchino S, Kellum JA, Bellomo R, Doig GS, Morimatsu H, Morgera S, Schetz M, Tan I, Bouman C, Macedo E, Gibney N, Tolwani A, Ronco C (2005). Beginning and ending supportive therapy for the kidney (BEST Kidney) investigators, acute renal failure in critically ill patients: a multinational, multicenter study. JAMA.

[2] Uchino S, Fealy N, Baldwin I, Morimatsu H, Bellomo R (2003). Continuous is not continuous: the incidence and impact of circuit “down-time” on uraemic control during continuous veno-venous haemofiltration. Intensive Care Med.

[3] Wu MY, Hsu YH, Bai CH, Lin YF, Wu CH, Tam KW (2012). Regional citrate versus heparin anticoagulation for continuous renal replacement therapy: a meta-analysis of randomized controlled trials. Am J Kidney Dis.

[4] Mehta RL, McDonald BR, Aguilar MM, Ward DM (1990). Regional citrate anticoagulation for continuous arteriovenous hemodialysis in critically ill patients. Kidney Int.

[5] Mehta RL, McDonald BR, Ward DM (1991). Regional citrate anticoagulation for continuous arteriovenous hemodialysis. Contrib Nephrol.

[6] Palsson R, Niles JL (1999). Regional citrate anticoagulation in continuous venovenous hemofiltration in critically ill patients with a high risk of bleeding. Kidney Int.

[7] Kutsogiannis DJ, Mayers I, Chin WD, Gibney RT (2000). Regional citrate anticoagulation in continuous venovenous hemodiafiltration. Am J Kidney Dis.

[8] Tolwani AJ, Campbell RC, Schenk MB, Allon M, Warnock DG (2001). Simplified citrate anticoagulation for continuous renal replacement therapy. Kidney Int.

[9] Gabutti L, Marone C, Colucci G, Duchini F, Schönholzer C (2002). Citrate anticoagulation in continuous venovenous hemodiafiltration: a metabolic challenge. Intensive Care Med.

[10] Monchi M, Berghmans D, Ledoux D, Canivet JL, Dubois B, Damas P (2004). Citrate vs. heparin for anticoagulation in continuous venovenous hemofiltration: a prospective randomized study. Intensive Care Med.

[11] Bagshaw SM, Laupland KB, Boiteau PJ, Godinez-Luna T (2005). Is regional citrate superior to systemic heparin anticoagulation for continuous renal replacement therapy? A prospective observational study in an adult regional critical care system. J Crit Care.

[12] Kutsogiannis DJ, Gibney RT, Stollery D, Gao J (2005). Regional citrate versus systemic heparin anticoagulation for continuous renal replacement in critically ill patients. Kidney Int.

[13] Betjes MG, van Oosterom D, van Agteren M, van de Wetering J (2007). Regional citrate versus heparin anticoagulation during venovenous hemofiltration in patients at low risk for bleeding: similar hemofilter survival but significantly less bleeding. J Nephrol.

[14] Oudemans-van Straaten HM, Bosman RJ, Koopmans M, van der Voort PH, Wester JP, van der Spoel JI, Dijksman LM, Zandstra DF (2009). Citrate anticoagulation for continuous venovenous hemofiltration. Crit Care Med.

[15] Hetzel GR, Schmitz M, Wissing H, Ries W, Schott G, Heering PJ, Isgro F, Kribben A, Himmele R, Grabensee B, Rump LC (2011). Regional citrate versus systemic heparin for anticoagulation in critically ill patients on continuous venovenous haemofiltration: a prospective randomized multicentre trial. Nephrol Dial Transplant.

[16] Zhang Z, Hongying N (2012). Efficacy and safety of regional citrate anticoagulation in critically ill patients undergoing continuous renal replacement therapy. Intensive Care Med.

[17] Nurmohamed SA, Vervloet MG, Girbes AR, Ter Wee PM, Groeneveld AB (2007). Continuous venovenous hemofiltration with or without predilution regional citrate anticoagulation: a prospective study. Blood Purif.

[18] Nurmohamed SA, Jallah BP, Vervloet MG, Yldirim G, ter Wee PM, Groeneveld AB (2013). Continuous venovenous haemofiltration with citrate-buffered replacement solution is safe and efficacious in patients with a bleeding tendency: a prospective observational study. BMC Nephrol.

[19] Vervloet MG, Nurmohamed SA (2007). How do I use citrate-based CVVH in predilution. Neth J Crit Care.

[20] Saudan P, Niederberger M, De Seigneux S, Romand J, Pugin J, Perneger T, Martin PY (2006). Adding a dialysis dose to continuous hemofiltration increases survival in patients with acute renal failure. Kidney Int.

[21] Palevsky PM, Zhang JH, O′Connor TZ, Chertow GM, Crowley ST, Choudhury D, Finkel K, Kellum JA, Paganini E, Schein RM, Smith MW, Swanson KM, Thompson BT, Vijayan A, Watnick S, Star RA, Peduzzi P (2009). Intensity of renal support in critically ill patients with acute kidney injury. N Engl J Med 2008, 359:7–20. Erratum in. N Engl J Med.

[22] Bellomo R, Cass A, Cole L, Finfer S, Gallagher M, Lo S, McArthur C, McGuinness S, Myburgh J, Norton R, Scheinkestel C, Su S (2009). Intensity of continuous renal-replacement therapy in critically ill patients. N Engl J Med.

[23] Verma AK, Levine M, Shalansky SJ, Carter CJ, Kelton JG (2003). Frequency of heparin-induced thrombocytopenia in critical care patients. Pharmacotherapy.

[24] Trehel-Tursis V, Louvain-Quintard V, Zarrouki Y, Imbert A, Doubine S, Stéphan F (2012). Clinical and biologic features of patients suspected or confirmed to have heparin-induced thrombocytopenia in a cardiothoracic surgical ICU. Chest.

[25] Joannidis M, Kountchev J, Rauchenzauner M, Schusterschitz N, Ulmer H, Mayr A, Bellmann R (2007). Enoxaparin vs. unfractionated heparin for anticoagulation during continuous veno-venous hemofiltration: a randomized controlled crossover study. Intensive Care Med.

[26] Zhang Z, Ni H, Lu B (2012). Variables associated with circuit life span in critically ill patients undergoing continuous renal replacement therapy: a prospective observational study. ASAIO.

[27] Levine MN, Hirsh J, Gent M, Turpie AG, Cruickshank M, Weitz J, Anderson D, Johnson M (1994). A Randomized trial comparing activated thromboplastin time with heparin assay in patients with acute venous thromboembolism requiring large daily doses of heparin. Arch Intern Med.

[28] Uprichard J, Manning RA, Laffan MA (2010). Monitoring heparin anticoagulation in the acute phase response. Br J Haematol.

[29] Aarab R, van Es J, de Pont AC, Vroom MB, Middeldorp S (2013). Monitoring of unfractionated heparin in critically ill patients. Neth J Med.

[30] Kidney Disease (2012). Improved Global Outcomes (KDIGO) Acute Kidney Injury Working Group. KDIGO Clinical Practice Guidelines for Acute Kidney Injury. Kidney Int.

