# Supplementary material for: Citrate anticoagulation versus systemic heparinisation in continuous venovenous hemofiltration in critically ill patients with acute kidney injury: a multi-center randomized clinical trial
Source: Crit Care. 2014 Aug 16;18(4):472. doi: 10.1186/s13054-014-0472-6 (PMC4161888; doi:10.1186/s13054-014-0472-6)
Supplement: Additional file 1: — Composition of replacement fluids and calcium pump settings. Composition of the replacement fluids used, the rate of infusion of replacement solution coupled to the blood flow and the protocol for calcium pump adjustments during citrate-based continuous venovenous haemofiltration (CVVH). [file 13054_2014_472_MOESM1_ESM.docx]

**Composition of replacement fluids**

BH504 HF32bic HFCitPre

Sodium 140 140 139.9

Potassium 1.5 2.0 3.0

Magnesium 0.5 0.5 0.5

Calcium 1.5 1.75 -

Chloride 103 11.5 104

Glucose 11.0 1.0 5.0

Citrate - - 39.9

Bicarbonate - 32 -

Lactate 42.0 3.0 -

In mmol/L

**Calcium pump adjustments during citrate CVVH**

Table 1. Initial pump settings (systemic ionised calcium of 1.0 – 1.1 mmol/l)

Blood pump (mL/min) Citrate-substitution flow (mL/hr) Calcium pump (mL/hr)

140 1900 9.5

160 2100 10.5

180 2400 12

200 2700 13.5

Table 2: Low calcium: 0.9 – 1.0 mmol/l.

Blood pump (ml/min) Citrate-substitution flow (ml/hr) Calcium pump (ml/hr)

140 1900 11

160 2100 12.5

180 2400 14

200 2700 16

Table 3: Low calcium: 0.8 – 0.9 mmol/l.

Blood pump (ml/min) Citrate-substitution flow (ml/hr) Calcium pump (ml/hr)

140 1900 15

160 2100 16.5

180 2400 18

200 2700 20

Table 4: High calcium

Blood pump (ml/min) Citrate-substitution flow (ml/hr) Calcium pump (ml/hr)

140 1900 7.5

160 2100 8.5

180 2400 10

200 2700 11
