# Supplementary material for: Citrate anticoagulation versus systemic heparinisation in continuous venovenous hemofiltration in critically ill patients with acute kidney injury: a multi-center randomized clinical trial
Source: Crit Care. 2014 Aug 16;18(4):472. doi: 10.1186/s13054-014-0472-6 (PMC4161888; doi:10.1186/s13054-014-0472-6)
Supplement: Additional file 2: — List of participating centres and number of patients included. The number of patients included by participating centres. [file 13054_2014_472_MOESM2_ESM.docx]

Additional file 2.

List of participating centers and number of patients included

Number of patients included

Rijnstate hospital, Arnhem 39

Hagahospital, Den Haag 26

VU University medical center, Amsterdam 25

Spaarne hospital, Hoofddorp 19

Medical center Alkmaar 12

Sint Lucas Andreas hospital, Amsterdam 7

Elisabeth hospital, Tilburg 3

Rijnland hospital, Leiderdorp 3

Slotervaart hospital, Amsterdam 3

University medical center Groningen, Groningen 2
