# Supplementary material for: Citrate anticoagulation versus systemic heparinisation in continuous venovenous hemofiltration in critically ill patients with acute kidney injury: a multi-center randomized clinical trial
Source: Crit Care. 2014 Aug 16;18(4):472. doi: 10.1186/s13054-014-0472-6 (PMC4161888; doi:10.1186/s13054-014-0472-6)
Supplement: Additional file 3: — Consort diagram. Consort diagram demonstrating eligible patients. Also, crossovers between anticoagulation groups are listed. [file 13054_2014_472_MOESM3_ESM.doc]

Screened for eligibility;
patients anticipated to require CVVH (n=2300)

Excluded (n=2161)

- Not meeting inclusion criteria

-Increased bleeding risk^1^ (n=1297)
 -Therapeutic anticoagulation (n=432)
 -Others^2^ (n=324)

- Declined to participate (n=108)

Randomized (n=139)

Allocated to citrate (n=66)

- Received allocated intervention (n=66)

Allocated to heparin (n=73)

- Received allocated intervention (n=73)

Discontinued (n=5):

- Citrate accumulation (n=4)
- Miscellaneous (n=1)

Cross over to heparin (n=4)

Discontinued (n=24):

- Bleeding episode (n=8)
- HITT (n=6)
- Frequent circuit failure (n=7)
- Miscellaneous (n=3)

Cross over to citrate (n=24)

Within 72hrs (n=9)

Per protocol citrate (n=61)

Per protocol heparin (n=49)

CVVH = continuous venovenous hemofiltration, HIT = heparin-induced thrombocytopenia and thrombosis.

^1^Defined as a platelet count of less than 40 x10^9^/L, an activated partial thromboplastin time (aPTT) of longer than 60 seconds, a prothrombin time-international normalised ratio (PT-INR) of more than 2.0 or a recent major bleeding). ^2^Including known HIT, not meeting age requirements.
