# Supplementary material for: Citrate anticoagulation versus systemic heparinisation in continuous venovenous hemofiltration in critically ill patients with acute kidney injury: a multi-center randomized clinical trial
Source: Crit Care. 2014 Aug 16;18(4):472. doi: 10.1186/s13054-014-0472-6 (PMC4161888; doi:10.1186/s13054-014-0472-6)
Supplement: Additional file 4: — Mortality in subgroups at 28 days. Mortality at 28 days for citrate and heparin in the following subgroups: age (higher versus lower than median), acute physiology and chronic health evaluation (APACHE) II scores (higher versus lower than the median), prescribed dose (higher or lower than the recommended 20 mL/kg/h), sepsis versus non-sepsis and circulatory or respiratory failure at ICU admission. [file 13054_2014_472_MOESM4_ESM.doc]

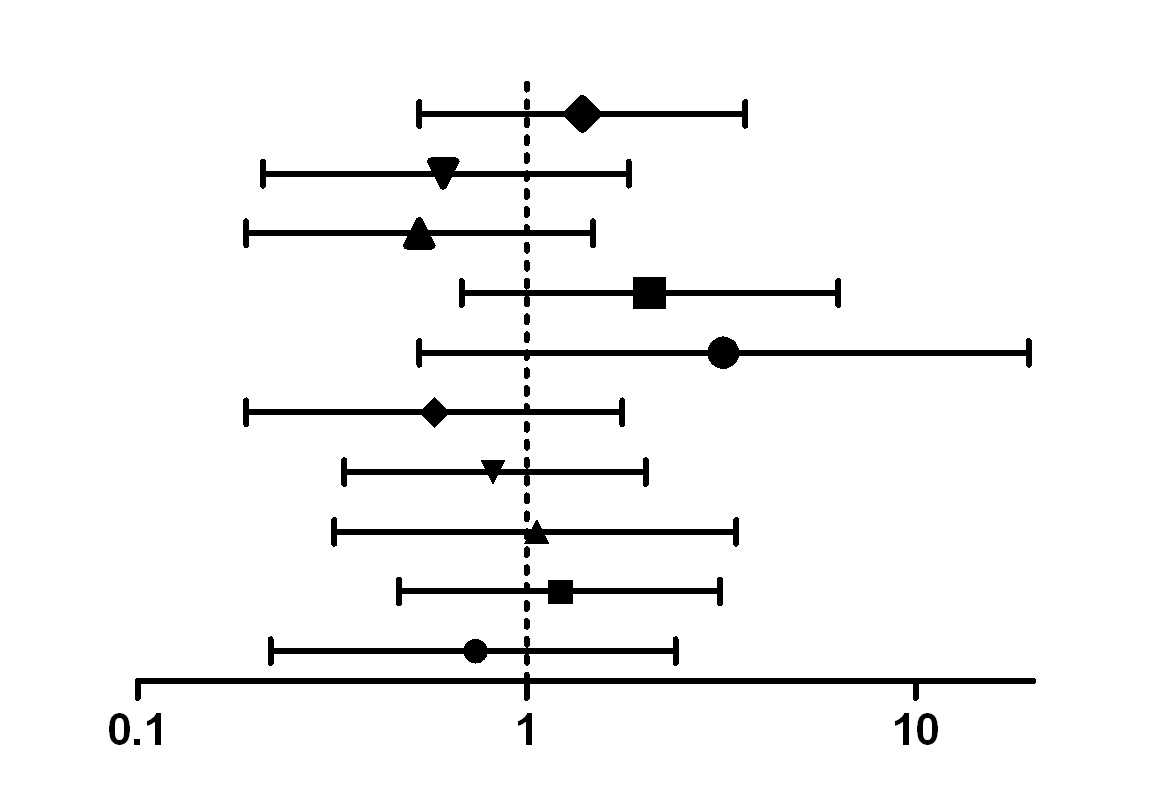


All patients Citrate Heparin Odds ratio (95% CI)

Age >67 yrs 29/68 (43) 15/32 (47) 14/36 (39) 1.39 (0.53-3.64)

≤67 yrs 18/69 (26) 7/33 (21) 11/36 (31) 0.61 (0.31-1.83)

APACHE score^1^ >24 28/64 (44) 9/26 (35) 19/38 (50) 0.53 (0.19-1.48)

≤24 18/73 (25) 12/39 (31) 6/34 (18) 2.07 (0.68-6.32)

ICU admission Circulatory failure 10/28 (36) 8/18 (44) 2/10 (20) 3.2 (0.53-19.50) Respiratory failure 23/68 (34) 6/23 (26) 17/45 (38) 0.58 (0.19-1.76)

Sepsis yes 30/84 (36) 13/39 (33) 17/45 (38) 0.82 (0.34-2.02) no 16/51 (31) 8/25 (32) 8/26 (31) 1.06 (0.32-3.46)

Prescribed dose>20 mL/kg/h 26/77 (34) 13/36 (36) 13/41 (32) 1.22 (0.47-3.14)

≤20 mL/kg/h 16/51 (31) 7/25 (28) 9/26 (35) 0.74 (0.22-2.41)

Citrate better Heparin better

Number of deaths/number of patients (%), ^1^ APACHE II scores at admission, CI = confidence interval.
